# Supplementary material for: Effective methods for bulk RNA-seq deconvolution using scnRNA-seq transcriptomes
Source: Genome Biol. 2023 Aug 1;24:177. doi: 10.1186/s13059-023-03016-6 (PMC10394903; doi:10.1186/s13059-023-03016-6)
Supplement: Supplementary file 1 — Additional file 1: Supplementary Figures S1-S7. Includes the presentation of detailed analyses and comparisons of assays and methods. [file 13059_2023_3016_MOESM1_ESM.pdf]

SUPPLEMENTARY FIGURES

Figure S1. (**A**) UMAP of merged scRNA-seq profiles of the 6 cell mixtures with 21 clusters predicted using default parameters revealed groups of adjacent clusters. (**B**) The expression profiles of some clusters were significantly correlated and supervised bi-clustering using a 2000-marker gene set identified cliques of correlated clusters that were also adjacent in the UMAP. Merging 3 cluster cliques, shown as regrouped clusters (top), reduced the total number of clusters to 6. (**C**) Deconvolution using the original 21 clusters, and (**D**) the reduced set of 6 clusters produced markedly different deconvolution accuracy evaluations. Here, the gold standard was estimated from scRNA-seq analysis for fairness since there is no 1-to-1 mapping between 21 clusters and 6 cell types. Accuracy was evaluated using Pearson correlations (P) and RMSE (E).

Figure S2. Matched breast cancer samples were profiled fresh or after cryopreservation by scRNA-seq. Confirming our observations from the analysis of kidney samples (Figure 4C), the accuracy of tested deconvolution methods was lower when using profiles of cryopreserved tissues. However, SQUID composition estimates were the most accurate based on fresh profiles and were nearly unaffected by tissue preservation.

Figure S3. Analogous to Figure 4A, deconvolution accuracy on concurrently profiled tissues suggested that DWLS outperforms other published methods irrespective of normalization. However, SQUID estimates were the most accurate on every dataset. Note that accuracy estimates reported in Figure 5 were based on cross-validation test errors, while estimates reported here and in Figure 4 did not use cross validation.

Figure S4. Analogous to Figure 4B, normalization strategies altered deconvolution accuracy estimates, but the top-performing methods outperformed other methods in nearly all tests, irrespective of normalization. In particular, the accuracy of SQUID estimates was nearly unaffected by the normalization strategy used.

Figure S5. Analogous to Figure 4C, deconvolution accuracies were estimated for fresh, methanol-fixed, and cryopreservation after warm and cold dissociation of kidney tissues. Deconvolution accuracy was lower for profiles of cryopreserved kidney tissues than for profiles of fresh or methanol-preserved tissues. However, SQUID's accuracy was high irrespective of the tissue preservation technique used.

Figure S6. Analogous to Figures 3B and 4, we present a detailed comparison between predicted abundance estimates by deconvolution and gold standard abundance estimates for the normalization strategy with the lowest RMSE for each dataset. In addition, we included a comparison of Bisque when using all genes and after marker gene selection. These were excluded from other plots because the implementation of Bisque doesn't allow for optimizing data normalization methods; axes are in $\log_{10}$ scale.

Figure S7. Our strategy for counting cell types using flow. All flow samples were prepared and analyzed in triplicate. For each of the six mixtures (1–6), three flow samples (1A–1C, 2A–2C, etc.) containing one Hoechst-stained cell line (either T47D, BT474, or MCF7), one Syto 82-stained cell line (either Jurkat, THP1, or hMSC), and four unstained cell lines at identical proportions were generated. Single-stained cells from these samples represented the proportion of that cell line in the corresponding mixture. This strategy was developed to avoid spectral overlap and to increase our ability to accurately quantify positive cells.

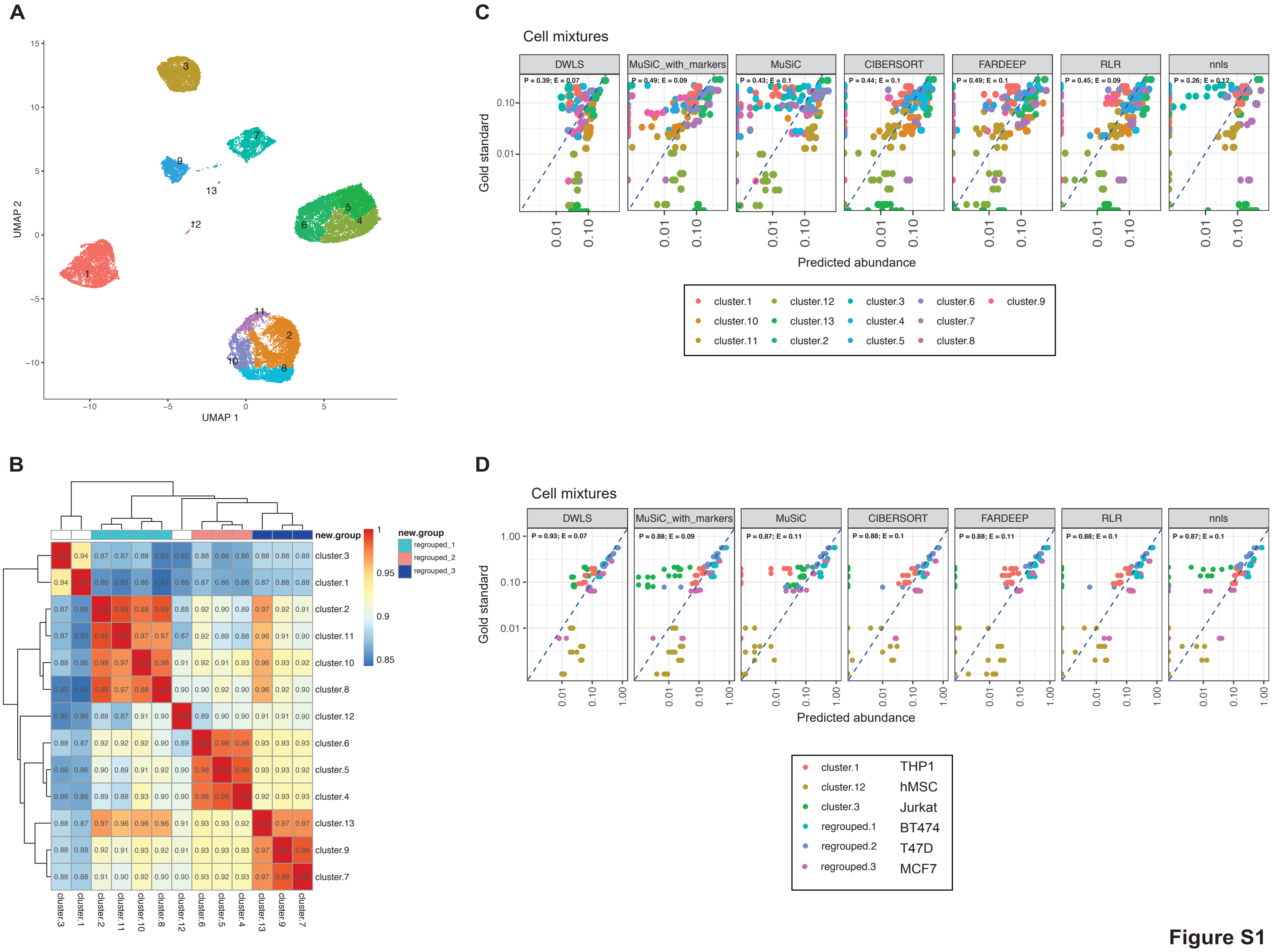
A
UMAP 1
UMAP 2
B
new.group
regrouped_1
regrouped_2
regrouped_3
cluster.3
cluster.1
cluster.2
cluster.11
cluster.10
cluster.8
cluster.12
cluster.6
cluster.5
cluster.4
cluster.13
cluster.9
cluster.7
C
Cell mixtures
DWLS
MuSiC_with_markers
MuSiC
CIBERSORT
FARDEEP
RLR
nnls
P = 0.39; E = 0.07
P = 0.49; E = 0.09
P = 0.43; E = 0.1
P = 0.44; E = 0.1
P = 0.49; E = 0.1
P = 0.45; E = 0.09
P = 0.26; E = 0.12
Gold standard
Predicted abundance
cluster.1
cluster.10
cluster.11
cluster.12
cluster.13
cluster.2
cluster.3
cluster.4
cluster.5
cluster.6
cluster.7
cluster.8
cluster.9
D
Cell mixtures
DWLS
MuSiC_with_markers
MuSiC
CIBERSORT
FARDEEP
RLR
nnls
P = 0.93; E = 0.07
P = 0.88; E = 0.09
P = 0.87; E = 0.11
P = 0.88; E = 0.1
P = 0.88; E = 0.11
P = 0.88; E = 0.1
P = 0.87; E = 0.1
Gold standard
Predicted abundance
cluster.1 THP1
cluster.12 hMSC
cluster.3 Jurkat
regrouped.1 BT474
regrouped.2 T47D
regrouped.3 MCF7

Figure S1

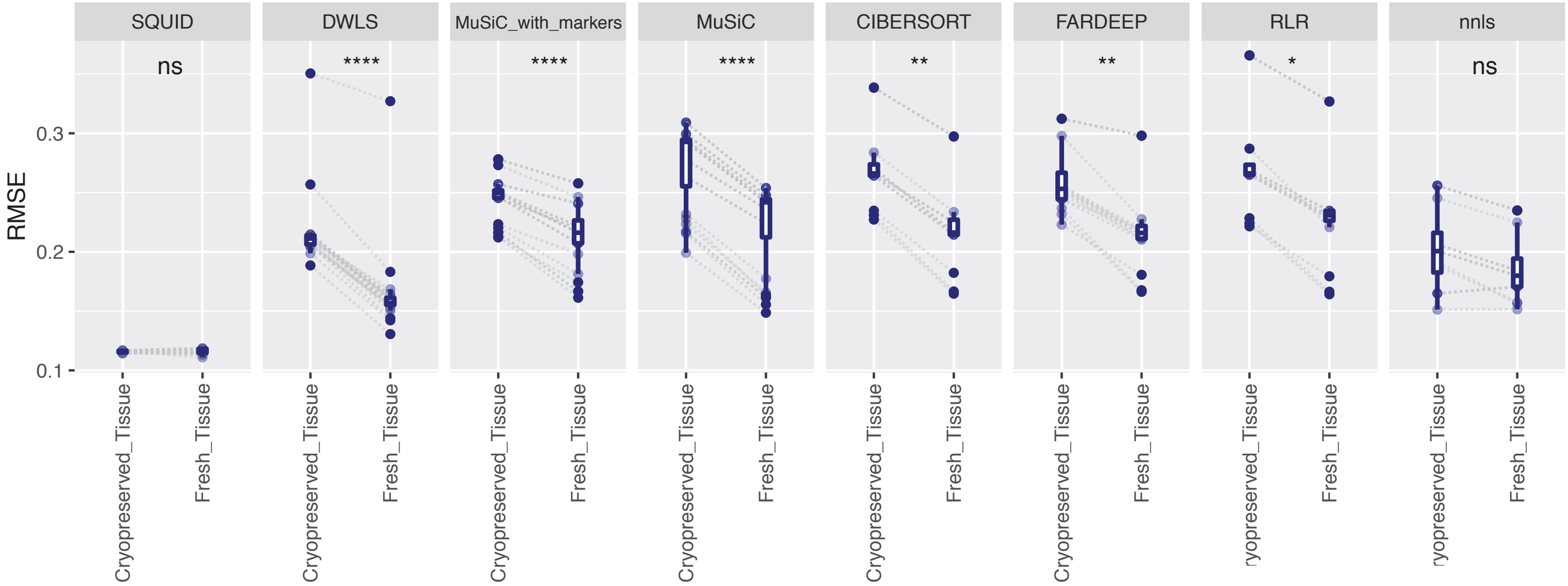
SQUID
DWLS
MuSiC_with_markers
MuSiC
CIBERSORT
FARDEEP
RLR
nnls
ns
****
****
****
**
**
*
ns
RMSE
0.3
0.2
0.1
Cryopreserved_Tissue
Fresh_Tissue

Figure S2

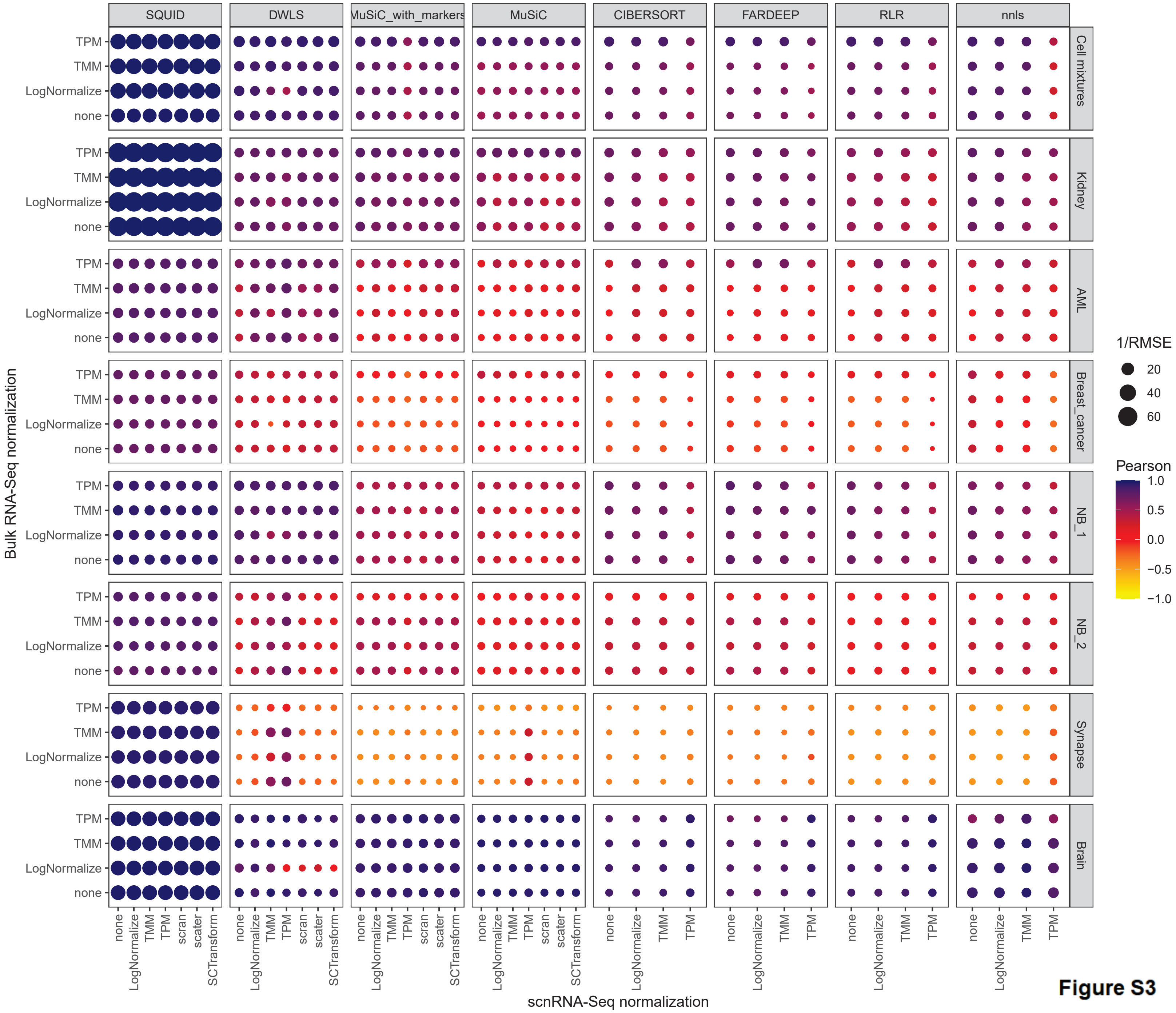
SQUID
DWLS
MuSiC_with_markers
MuSiC
CIBERSORT
FARDEEP
RLR
nnls
Cell mixtures
Kidney
AML
Breast_cancer
NB_1
NB_2
Synapse
Brain
TPM
TMM
LogNormalize
none
scran
scater
SCTransform
Bulk RNA-Seq normalization
scnRNA-Seq normalization
1/RMSE
20
40
60
Pearson
1.0
0.5
0.0
−0.5
−1.0

Figure S3

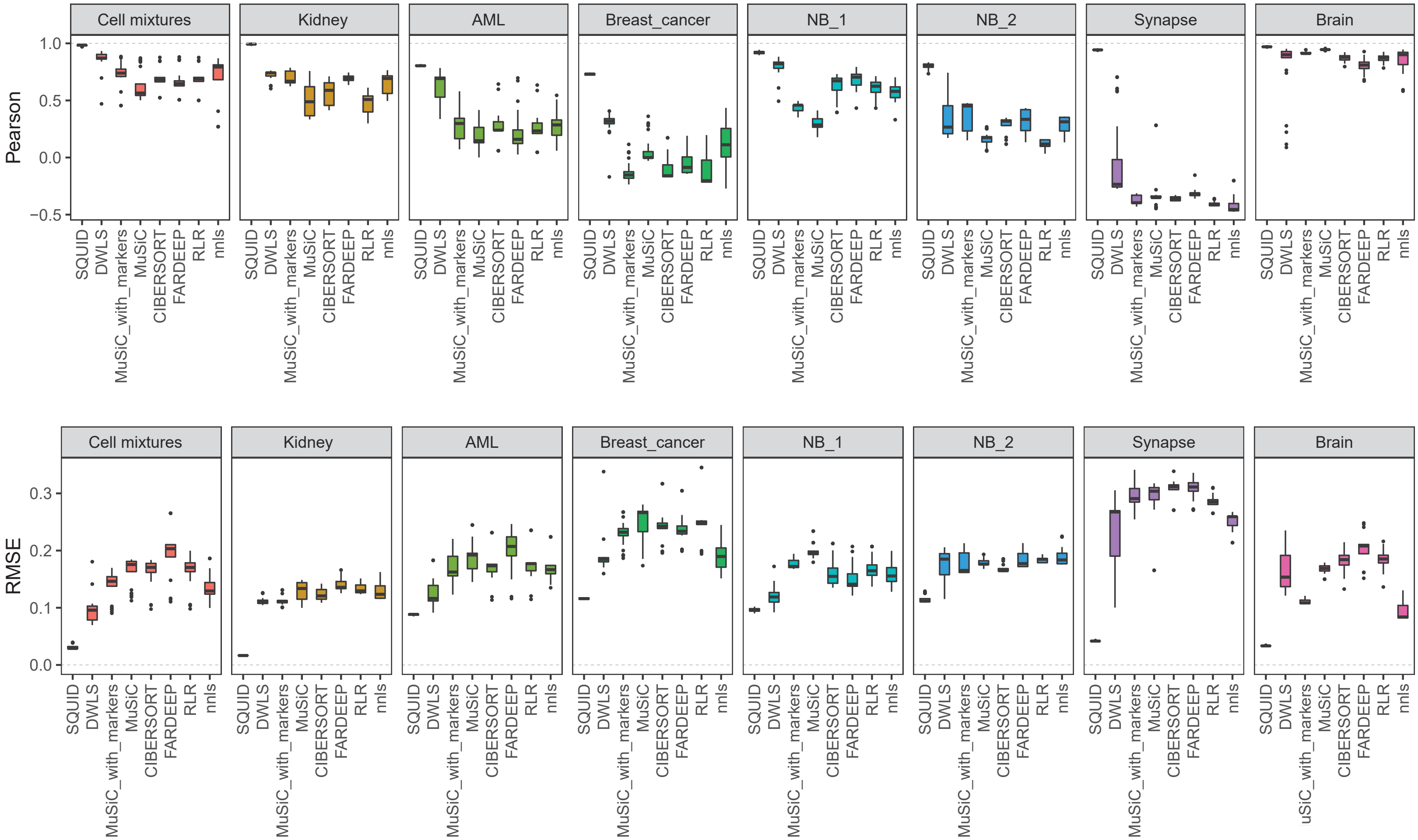
Cell mixtures
Kidney
AML
Breast_cancer
NB_1
NB_2
Synapse
Brain
Pearson
1.0
0.5
0.0
−0.5
SQUID
DWLS
MuSiC_with_markers
MuSiC
CIBERSORT
FARDEEP
RLR
nnls
RMSE
0.3
0.2
0.1
0.0
uSiC_with_markers

Figure S4

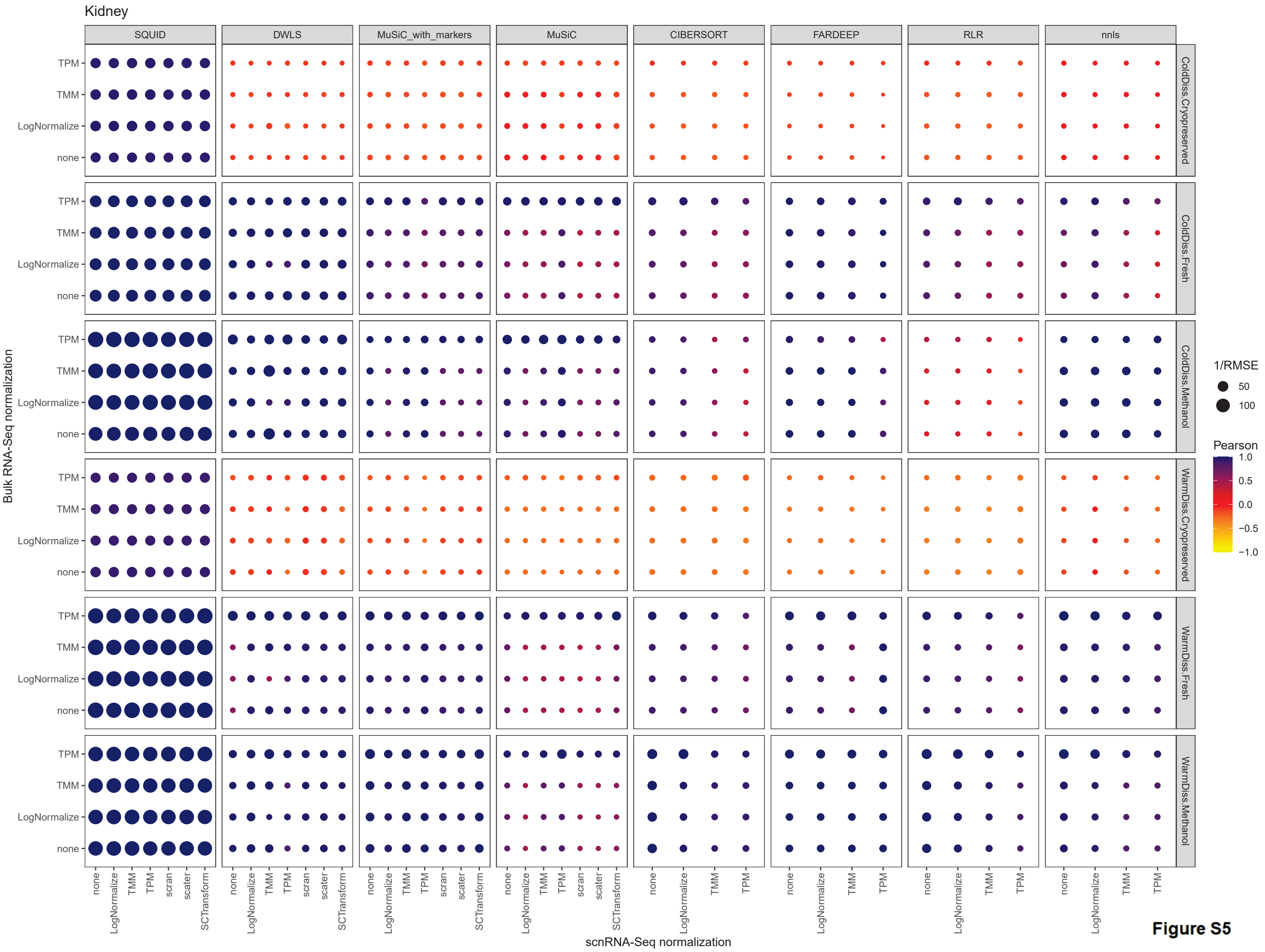

Kidney
SQUID
DWLS
MuSiC_with_markers
MuSiC
CIBERSORT
FARDEEP
RLR
nnls
ColdDiss.Cryopreserved
ColdDiss.Fresh
ColdDiss.Methanol
WarmDiss.Cryopreserved
WarmDiss.Fresh
WarmDiss.Methanol
TPM
TMM
LogNormalize
none
scran
scater
SCTransform
Bulk RNA-Seq normalization
scnRNA-Seq normalization
1/RMSE
50
100
Pearson
1.0
0.5
0.0
−0.5
−1.0

Figure S5

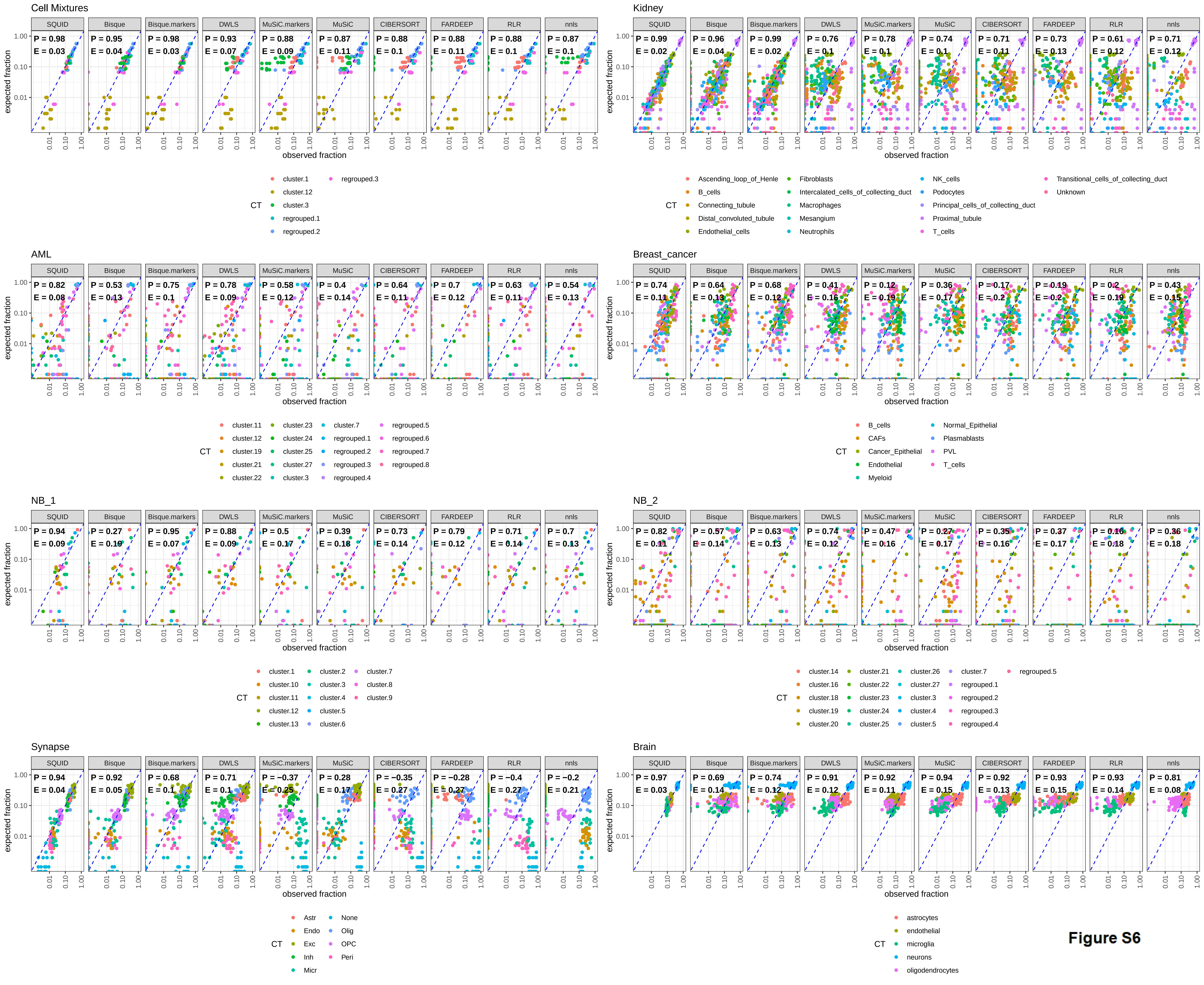
Cell Mixtures
SQUID P = 0.98 E = 0.03
Bisque P = 0.95 E = 0.04
Bisque.markers P = 0.98 E = 0.03
DWLS P = 0.93 E = 0.07
MuSiC.markers P = 0.88 E = 0.09
MuSiC P = 0.87 E = 0.11
CIBERSORT P = 0.88 E = 0.1
FARDEEP P = 0.88 E = 0.11
RLR P = 0.88 E = 0.1
nnls P = 0.87 E = 0.1
expected fraction
observed fraction
CT
cluster.1
cluster.12
cluster.3
regrouped.1
regrouped.2
regrouped.3
Kidney
SQUID P = 0.99 E = 0.02
Bisque P = 0.96 E = 0.04
Bisque.markers P = 0.99 E = 0.02
DWLS P = 0.76 E = 0.1
MuSiC.markers P = 0.78 E = 0.1
MuSiC P = 0.74 E = 0.1
CIBERSORT P = 0.71 E = 0.11
FARDEEP P = 0.73 E = 0.13
RLR P = 0.61 E = 0.12
nnls P = 0.71 E = 0.12
CT
Ascending_loop_of_Henle
B_cells
Connecting_tubule
Distal_convoluted_tubule
Endothelial_cells
Fibroblasts
Intercalated_cells_of_collecting_duct
Macrophages
Mesangium
Neutrophils
NK_cells
Podocytes
Principal_cells_of_collecting_duct
Proximal_tubule
T_cells
Transitional_cells_of_collecting_duct
Unknown
AML
SQUID P = 0.82 E = 0.08
Bisque P = 0.53 E = 0.13
Bisque.markers P = 0.75 E = 0.1
DWLS P = 0.78 E = 0.09
MuSiC.markers P = 0.58 E = 0.12
MuSiC P = 0.4 E = 0.14
CIBERSORT P = 0.64 E = 0.11
FARDEEP P = 0.7 E = 0.12
RLR P = 0.63 E = 0.11
nnls P = 0.54 E = 0.13
CT
cluster.11
cluster.12
cluster.19
cluster.21
cluster.22
cluster.23
cluster.24
cluster.25
cluster.27
cluster.3
cluster.7
regrouped.1
regrouped.2
regrouped.3
regrouped.4
regrouped.5
regrouped.6
regrouped.7
regrouped.8
Breast_cancer
SQUID P = 0.74 E = 0.11
Bisque P = 0.64 E = 0.13
Bisque.markers P = 0.68 E = 0.12
DWLS P = 0.41 E = 0.16
MuSiC.markers P = 0.12 E = 0.19
MuSiC P = 0.36 E = 0.17
CIBERSORT P = 0.17 E = 0.2
FARDEEP P = 0.19 E = 0.2
RLR P = 0.2 E = 0.19
nnls P = 0.43 E = 0.15
CT
B_cells
CAFs
Cancer_Epithelial
Endothelial
Myeloid
Normal_Epithelial
Plasmablasts
PVL
T_cells
NB_1
SQUID P = 0.94 E = 0.09
Bisque P = 0.27 E = 0.19
Bisque.markers P = 0.95 E = 0.07
DWLS P = 0.88 E = 0.09
MuSiC.markers P = 0.5 E = 0.17
MuSiC P = 0.39 E = 0.18
CIBERSORT P = 0.73 E = 0.14
FARDEEP P = 0.79 E = 0.12
RLR P = 0.71 E = 0.14
nnls P = 0.7 E = 0.13
CT
cluster.1
cluster.10
cluster.11
cluster.12
cluster.13
cluster.2
cluster.3
cluster.4
cluster.5
cluster.6
cluster.7
cluster.8
cluster.9
NB_2
SQUID P = 0.82 E = 0.11
Bisque P = 0.57 E = 0.14
Bisque.markers P = 0.63 E = 0.13
DWLS P = 0.74 E = 0.12
MuSiC.markers P = 0.47 E = 0.16
MuSiC P = 0.27 E = 0.17
CIBERSORT P = 0.35 E = 0.16
FARDEEP P = 0.37 E = 0.17
RLR P = 0.16 E = 0.18
nnls P = 0.36 E = 0.18
CT
cluster.14
cluster.16
cluster.18
cluster.19
cluster.20
cluster.21
cluster.22
cluster.23
cluster.24
cluster.25
cluster.26
cluster.27
cluster.3
cluster.4
cluster.5
cluster.7
regrouped.1
regrouped.2
regrouped.3
regrouped.4
regrouped.5
Synapse
SQUID P = 0.94 E = 0.04
Bisque P = 0.92 E = 0.05
Bisque.markers P = 0.68 E = 0.1
DWLS P = 0.71 E = 0.1
MuSiC.markers P = −0.37 E = 0.25
MuSiC P = 0.28 E = 0.17
CIBERSORT P = −0.35 E = 0.27
FARDEEP P = −0.28 E = 0.27
RLR P = −0.4 E = 0.27
nnls P = −0.2 E = 0.21
CT
Astr
Endo
Exc
Inh
Micr
None
Olig
OPC
Peri
Brain
SQUID P = 0.97 E = 0.03
Bisque P = 0.69 E = 0.14
Bisque.markers P = 0.74 E = 0.12
DWLS P = 0.91 E = 0.12
MuSiC.markers P = 0.92 E = 0.11
MuSiC P = 0.94 E = 0.15
CIBERSORT P = 0.92 E = 0.13
FARDEEP P = 0.93 E = 0.15
RLR P = 0.93 E = 0.14
nnls P = 0.81 E = 0.08
CT
astrocytes
endothelial
microglia
neurons
oligodendrocytes

Figure S6

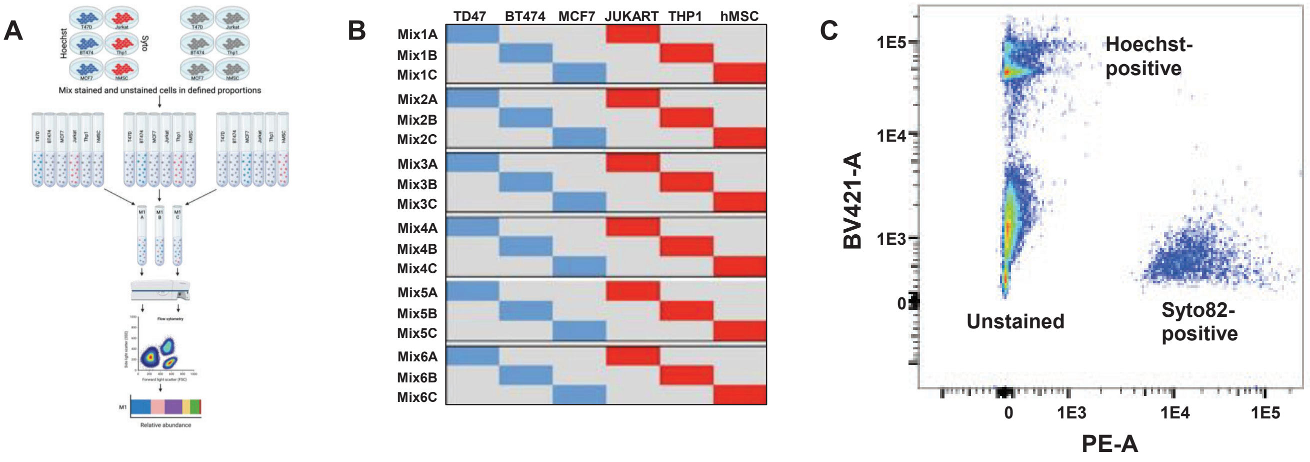
A
Hoechst
Syto
T47D
Jurkat
BT474
Thp1
MCF7
hMSC
Mix stained and unstained cells in defined proportions
M1 A
M1 B
M1 C
Flow cytometry
Side light scatter (SSC)
Forward light scatter (FSC)
M1
Relative abundance
B
TD47
BT474
MCF7
JUKART
THP1
hMSC
Mix1A
Mix1B
Mix1C
Mix2A
Mix2B
Mix2C
Mix3A
Mix3B
Mix3C
Mix4A
Mix4B
Mix4C
Mix5A
Mix5B
Mix5C
Mix6A
Mix6B
Mix6C
C
BV421-A
1E5
1E4
1E3
0
PE-A
0
1E3
1E4
1E5
Hoechst-positive
Unstained
Syto82-positive

Figure S7
